# TYGS and LPSN: a database tandem for fast and reliable genome-based classification and nomenclature of prokaryotes

**DOI:** 10.1093/nar/gkab902

**Published:** 2021-10-11

**Authors:** Jan P Meier-Kolthoff, Joaquim Sardà Carbasse, Rosa L Peinado-Olarte, Markus Göker

**Affiliations:** Leibniz Institute DSMZ – German Collection of Microorganisms and Cell Cultures, Department of Bioinformatics and Databases, Inhoffenstrasse 7B, 38124 Braunschweig, Germany; Leibniz Institute DSMZ – German Collection of Microorganisms and Cell Cultures, Department of Bioinformatics and Databases, Inhoffenstrasse 7B, 38124 Braunschweig, Germany; Leibniz Institute DSMZ – German Collection of Microorganisms and Cell Cultures, Department of Bioinformatics and Databases, Inhoffenstrasse 7B, 38124 Braunschweig, Germany; Leibniz Institute DSMZ – German Collection of Microorganisms and Cell Cultures, Department of Bioinformatics and Databases, Inhoffenstrasse 7B, 38124 Braunschweig, Germany

## Abstract

Microbial systematics is heavily influenced by genome-based methods and challenged by an ever increasing number of taxon names and associated sequences in public data repositories. This poses a challenge for database systems, particularly since it is obviously advantageous if such data are based on a globally recognized approach to manage names, such as the International Code of Nomenclature of Prokaryotes. The amount of data can only be handled if accurate and reliable high-throughput platforms are available that are able to both comply with this demand and to keep track of all changes in an efficient and flexible way. The List of Prokaryotic names with Standing in Nomenclature (LPSN) is an expert-curated authoritative resource for prokaryotic nomenclature and is available at https://lpsn.dsmz.de. The Type (Strain) Genome Server (TYGS) is a high-throughput platform for accurate genome-based taxonomy and is available at https://tygs.dsmz.de. We here present important updates of these two previously introduced, heavily interconnected platforms for taxonomic nomenclature and classification, including new high-level facilities providing access to bioinformatic algorithms, a considerable expansion of the database content, and new ways to easily access the data.

## INTRODUCTION

The global prokaryotic diversity is estimated to encompass 0.8–1.6 million species (1) of which currently only about 1% (2) have a name that is validly published under the International Code of Nomenclature of Prokaryotes (ICNP) (3). Even though these estimates are likely to fluctuate, microbial life still remains largely unknown and unconnected to the only system of nomenclature accepted by the majority of microbiologists, the ICNP. But a continuous and systematic understanding of this large genetic and enzymatic reservoir is necessary to further elucidate the biological mechanisms affecting important topics such as the global geochemical cycles, public health and biotechnology.

For more than a century, microbial taxonomy is dedicated to the characterization, classification and nomenclature of microbial life. The data acquired through characterization (4), which presently significantly relies on genome-based approaches (5), are used to establish the classification of microbes. Classification in turn needs an internationally accepted system of nomenclature to unambiguously assign names to taxa (3). The cornerstone of the ICNP is the status of taxon names as being validly published; other names have no claim to recognition under this code (3).

Due to the fast-paced changes in prokaryotic nomenclature and the continued influx of newly proposed names, expert-curated and at least partially automated database systems are needed to keep track of these processes. One of the most influential and authoritative resources in this regard is the List of Prokaryotic names with Standing in Nomenclature (LPSN), which was first introduced more than two decades ago as a manually curated database (6) and maintained over the years (7). Since 2020 LPSN is located at https://lpsn.dsmz.de at the Leibniz Institute DSMZ – German Collection of Microorganisms and Cell Cultures (2). The move from the previous platform to the DSMZ was accompanied by the establishment of a database infrastructure, of automated data import routines and of a database-driven web interface (2). Pages for individual taxon names were equipped with information on etymology, type-strain deposits, 16S rRNA gene sequences in FASTA format, INSDC 16S rRNA gene accession numbers, taxonomic and nomenclatural status and many notes (2).

Taxonomic classification is currently strongly influenced by genome-scale data and related methods (8–15). The ever-increasing growth of genome data (16) demands technical solutions for efficient, reproducible and standardized taxonomic analyses. Comparisons with the genome sequences of type strains are mandatory when classifying novel strains (4). Hence, a reliable mapping between type strains, genome sequences and taxon names has to be established.

To address these requirements, the Type (Strain) Genome Server (TYGS) was previously introduced as a large database of curated type-strain genomes combined with selected metadata and facilities for conducting truly genome-based taxonomic analyses in a high-throughput setup (17). Queries to the TYGS database, located at https://tygs.dsmz.de, are made with one to several uploaded genome sequences. The results include genome-scale phylogenies and state-of-the-art estimates for species and subspecies boundaries for both user-provided sequences and automatically determined closest type genome sequences. Metadata for these are provided to facilitate the taxonomic exploration of the outcome, including nomenclature, synonymy and associated taxonomic literature (17).

In this study we present the current state of the heavily used TYGS and LPSN databases, including updates and improvements which were added since the last publications (2,17). The establishment of LPSN at the DSMZ allows for strongly interconnecting the two databases (2,17) to further increase their usability, a topic that is also addressed below.

## TYGS AND LPSN IN 2021

### The databases

**Figure 1.**
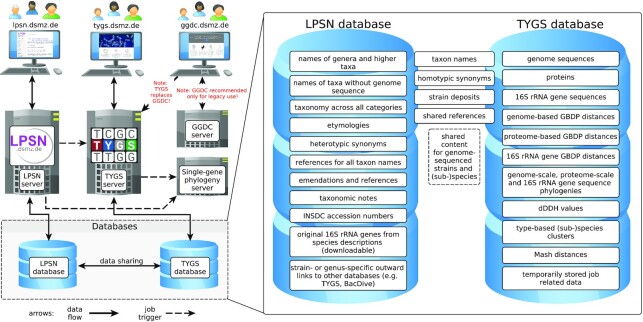
Left, dependencies of the TYGS and LPSN platforms and relationships to the legacy GGDC platform and to the DSMZ gene phylogeny server. Right, description of the most relevant (partially shared) contents of the TYGS and LPSN databases.

**Figure 2.**
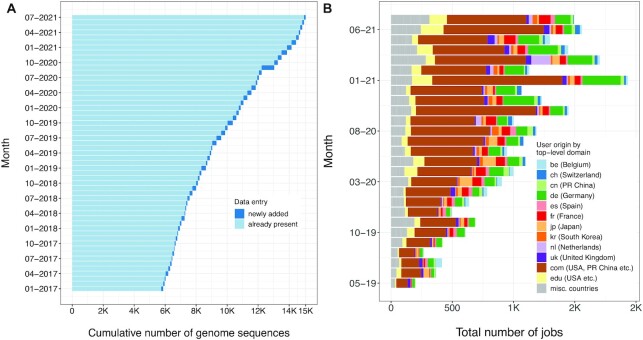
(**A**) statistics on the cumulative, non-linear growth of TYGS type-strain genome sequences per month. (**B**) non-cumulative number of TYGS job submissions per month since its establishment in 2019. Job submissions are grouped according to the top-level domain of the e-mail address included in each job submission. The top-level domain was matched to the country of origin wherever possible.

As of July 2021, the LPSN database (Figure 1) provides a large variety of expert-curated nomenclatural data, including >26k names of any category and >21k species names validly published under the ICNP (3). At the time of writing, the TYGS database (Figure 1) contains >15k type-strain genomes and was used by >3.7k different users worldwide for conducting >27k distinct analyses (Figure 2) involving 15M + genome comparisons.

The content of the TYGS and LPSN databases was introduced in previous publications (2,17) and is here only briefly recapitulated (Figure 1). The depicted database content reflects the focus of the two databases, which accordingly share many kinds of data.

The authority of a taxon name reflects its original publication and thus links nomenclature and scientific literature. Synonymy of names is another key component of taxonomic information to which LPSN allows comprehensive access. The nomenclatural type is the entity with which a taxon name is permanently associated (3). In the cases of prokaryotic species and subspecies, the nomenclatural type is the type strain. Cultures of type strains thus firmly connect taxon names and genome sequences.

Quality-checked (17) genome and proteome sequences of verified type strains are the starting point of TYGS analyses. Accurate intergenomic GBDP (Genome BLAST Distance Phylogeny) (18) distances and digital DNA:DNA hybridization (dDDH) values are calculated and stored in the TYGS database. Most of them are inferred well in advance of user requests to considerably accelerate the processing of these requests. The distances are used for phylogenetic inference and also form the basis for the dDDH method, one of the most widely used overall genome relatedness indices (OGRI) for *in silico* species delineation (18,19). Genome-scale and proteome-scale phylogenies, 16S rRNA gene trees and type-based clusterings at the species and subspecies levels are the data entries that represent the final step of the comprehensive genome-based taxonomic analyses available in the TYGS.

Analyses on the DSMZ single-gene phylogeny server (Figure 1) can be triggered by the TYGS as well as via LPSN. The server calculates pairwise similarities (20), multiple sequence alignments (21) and phylogenetic trees under the maximum-likelihood (22) and maximum parsimony (23) criteria from single-gene data sets as introduced previously (24). The DSMZ single-gene phylogeny server is also contacted by the TYGS on user request to infer single-gene phylogenies from a comprehensive set of 16S rRNA gene sequences that may include closely related but not yet genome-sequenced type strains.

### New kinds of TYGS data and new TYGS facilities

The initial release of the TYGS determined a set of type strains most closely related to a respective user sequence by using only 16S rRNA gene sequence distances (17). As not all user sequences contain an extractable 16S rRNA gene, the most closely related type-strain genomes are now also determined using intergenomic Mash distances (25), a fast initial approximation of intergenomic relatedness. The set of type-strain genomes having the smallest MASH distances among the entire TYGS database is combined with those having the smallest 16S rRNA gene distances. This approach also ensures that the most closely related type strains are chosen if the user genome sequence belongs to a group of organisms for which the 16S rRNA gene sequence does not properly resolve the phylogenetic relationships. However, the usage of 16S rRNA gene sequences remains highly relevant for detecting closely related type strains that have not yet been genome-sequenced.

Mash distances are now also used to split job submissions that contain query genomes that are only remotely related. If otherwise such an analysis would result in the selection of distinct sets of closest type strain genomes and trigger taxonomically useless calculations. Subdivided submissions, if any, are accordingly indicated on the job confirmation page. This feature further improves the genome-based taxonomic analyses by determining efficient type strain selection in a variety of data situations.

For very diverse datasets of strains, the average branch support even of a genome-scale phylogeny and even for a strain selection restricted as described above, might be too low when based on nucleotide sequences. We thus added the option to conduct an additional GBDP analysis using amino-acid sequences under the recommended settings (26), which automatically becomes available on the TYGS result page in case of insufficient support.

It should be noted that the Genome-to-Genome Distance Calculator (GGDC) preceded the TYGS and is still one of the most popular online tools for the calculation of dDDH values for *in silico* (sub-)species delineation (18,19). Although the current GGDC version, 3.0, also incorporates several new features and optimizations from the previous years, the database-driven TYGS offers many more facilities, overall. Although GGDC and TYGS are equally reliable regarding species delineation, GGDC users are strongly advised to switch to the TYGS. In rare cases, the GGDC 3.0 can still be useful for specialized analyses but since a larger file upload cap can usually be requested via the TYGS feedback form, the TYGS is certainly the more comprehensive platform since its initial release.

The series of publicly available pre-calculated TYGS result sets that can be accessed from the ‘Examples’ menu item on the TYGS main page demonstrates the new facilities listed above. All TYGS results are now also available within a compact, publication-ready PDF report.

Each TYGS result usually includes links to external sites such as Bac*Dive* (27) and GenBank to provide further information on each used type strain. As a result of the move of LPSN to DSMZ, the preferred names of each type strain listed on the respective TYGS result page now link to the specific LPSN page of that species. Links to the Bac*Dive* database now point to the specific strain page whenever possible.

### Expansion of the scope

**Figure 3.**
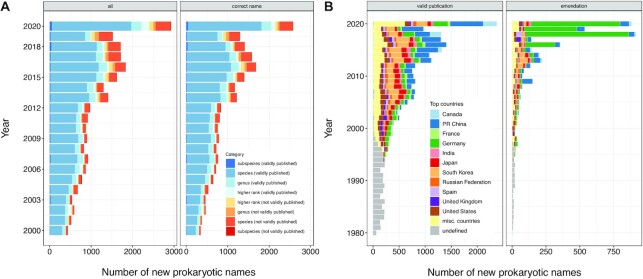
(**A**) Number of new prokaryotic names included in LPSN grouped by year and taxonomic category and nomenclatural status (shades of blue, validly published under the ICNP; shades of red, not validly published). Left, all names; right, names currently regarded as correct name (3) in LPSN. (**B**) Number of new prokaryotic names validly published under the ICNP and number of emendations of such names included in LPSN, grouped by year and country of origin of the taxonomic study. Left, number of validly published names; right, number of emendations of such names. The country of origin of each study represents the country of origin of the author indicated as corresponding author including an e-mail address.

The growth of the number of genome sequences in the TYGS database is shown in Figure 2 while the growth of the number of taxon names in the LPSN database is shown in Figure 3. As for names validly published under the ICNP (3), the increase of names in LPSN and accordingly of type-strain genome sequences in the TYGS simply mirrors the increase of the rate of valid published names per year. The number of validly published names per country and year reflects the well-known shift of taxonomic activity to East Asian nations (28). The relative and absolute number of emendations also shows an increasing trend, caused by the availability of genome sequences of type strains (8–11). More importantly, the overall number of names that become validly published per year shows an ever-increasing trend. One of the challenges of nomenclature-related databases is apparently the sheer increase of the volume of the data to be considered.

A major change regarding the selection of taxon names for inclusion in LPSN is the addition of names that are not validly published (*Candidatus* names and other names), as well as of names of *Cyanobacteria* validly published under the ICN (International Code of Nomenclature for algae, fungi and plants), in anticipation of the recent changes to the ICNP (29). Whereas they do not have standing in nomenclature, the addition of names to LPSN that have an effective publication but are not validly published had started at an early stage (6), although the names were not linked to the hierarchical classification.

An improved integration of not validly published names in LPSN reflects the inclusion of additional categories regulated under the rules of the ICNP (30) and the publication of lists of *Candidatus* names in the International Journal of Systematic and Evolutionary Microbiology (IJSEM) (31). This integration also has other advantages. For instance, many names with an effective publication outside IJSEM are validated at a later date anyway (3), hence their earlier consideration facilitates data analysis. Conversely, names supposed to be validly published may later on be recognized as not having met the necessary requirements (32). Moreover, not all taxonomist are aware of the need for validation (33). The storage of not validly published names in LPSN allows for conducting campaigns to increase the rate of validation. As of July 2021, almost 900 e-mails were sent to corresponding authors to inform them about names proposed by them that as yet have no claim to recognition under the ICNP (3), covering >1500 names.

An even more fundamental advantage of including not validly published names is that it adds clarity. If a name is shown on LPSN and explicitly marked as not being validly published, this leaves no doubt with regard to its status, as opposed to just not showing the name at all. Because they are not centrally collected in a journal such as IJSEM, the coverage of names by LPSN that have an effective publication but are not validly published under the code cannot be expected to be complete. LPSN attempts to focus on names that are in use, i.e. are found in the literature or in other databases. To improve the coverage, a web form for submission was added to LPSN that allows for anonymous suggestions of taxon names. Synonymy relationships between taxon names and assignments of child taxa to parent taxa (i.e, genera to families, families to orders etc.) can also be suggested. The inclusion criterion is the presence of an effective publication as defined by the ICNP (3).

### New kinds of information on LPSN

Apart from the constant addition of taxon names and associated information and the regular update of information provided by third parties, such as the risk-group assignments for species and subspecies (34), a variety of LPSN facilities and pages were established since the relaunch of the entire site at DSMZ (2). All of these pages are accessible through the LPSN navigation page.

Greatly expanded and regularly updated pages on Frequently Asked Questions, nomenclature, etymology, and taxonomy and systematics provide access to the relevant literature. A variety of pages provide more specific etymological information on bacterial names. The LPSN glossary now contains more entries and more cross references; key terms related to the rules of nomenclature (3) have been augmented to explain critical issues. Special pages on Requests for an Opinion and Judicial Opinions (32) are also intended to increase nomenclatural literacy.

The LPSN page on culture collections, which is augmented in parallel to the registering of type-strain deposits in LPSN, lists hundreds of biological resource centres. In addition to links and postal addresses the page now also contains country codes and WDCM (World Data Centre for Microorganisms) numbers to further ease the access to information on collections.

Comments on taxonomic terminology were added to LPSN for single taxon names where appropriate, as well as comments about the conditions under which a name would be regarded as the correct name if it is not currently so regarded by LPSN. The latter change is supposed to improve the understanding of the difference between nomenclature and classification, which may puzzle many microbiologists (35). LPSN now also includes a scoring based on genomic distances calculated by the TYGS (26). These comments highlight groups that may need a taxonomic revision.

### Improved access to the data

The LPSN CSV (comma-separated values) file became available for download once LPSN was established at DSMZ (2). An Excel file was previously offered by the PNU (Prokaryotic Nomenclature Up-to-date) service of DSMZ, which was superseded by the new LPSN. The LPSN CSV file needed to be downwards compatible with that Excel file as far as possible, hence it was and is intended to contain only the names of genera, species and subspecies validly published under the ICNP.

LPSN was augmented with an API (Application Programming Interface) to provide greater flexibility. The LPSN API is designed to be expandable in the future without creating backwards incompatibility. The LPSN API has a REST (Representational State Transfer) interface that allows for two kinds of searches and yields JSON (JavaScript Object Notation). Code examples and clients in two programming languages are provided inline.

Users are advised to register for the LPSN mailing list because forthcoming changes to CSV download file or API are announced on this list in order to avoid severe incompatibilities with previous formats. The LPSN record numbers as used in the CSV files and in the API are also visible on each page to ease comparisons.

The TYGS platform was similarly augmented with a REST API to allow users to download tabular results in the open interchange format JSON, phylogenetic results in either PhyloXML (36) or Newick format and scientific citations in BibTex format. Details on the API are available online and users are asked to follow the instructions on the TYGS API page on how and when to use the API.

## CONCLUSION

This update of the TYGS and LPSN platforms again underlines the need for an expert-curated nomenclatural database and for a database of verified type strain genomes, their metadata and subsequent facilities for taxonomic analysis, as well as an improved access to the data. Even though other platforms such as EzBioCloud (37) may provide access to a broader selection of ‘reference genomes’, including non-type strains, these are to the best of our knowledge frequently confused or misinterpreted as type strains and may thus lead to taxonomic misinterpretations. TYGS and LPSN, a synergistic pair of database-driven platforms, not only cover the core fields of (genome-based) taxonomy but also provide guidance especially for non-experts in a field in which one may have difficulties in choosing between a plethora of tools and data entries.

The calculation and storage of intergenomic distances and dDDH values provides for a high level of standardization within the TYGS database, with dDDH being one of the most widely used overall genome relatedness indices (OGRI) for *in silico* species delineation (18,19). As noted earlier, the dDDH approach was shown to outperform (18,19) the ‘average nucleotide identity’ (ANI) approach, which is also frequently used in scientific studies as an alternative OGRI index (5). More recently, ‘ANI’ was found to yield results that are inconsistent across its various implementations due to a lack of standardization (38).

While a recent attempt to modify the ICNP to allow for using genome sequences as nomenclatural type was unsuccessful (39), alternative solutions to the perceived underlying problem are currently actively debated (40). Independent of the outcome of these discussions, the expansion of the LPSN infrastructure demonstrates that it is already capable of dealing with taxon names that have a status under distinct codes of nomenclature. As expected for these two highly interconnected platforms, the broadening of the scope of names included in LPSN automatically also broadens the scope of genome sequences found in the TYGS.

## DATA AVAILABILITY

The provided TYGS data can be freely downloaded in various formats (e.g. CSV, XLS, JSON, PDF, SVG, PNG, PhyloXML, Newick and BibTeX) without restrictions, except that the origin of the data has to be properly cited (17) when used in other works. Information obtained from LPSN should also be properly cited (2). Registration is necessary to access the LPSN download files and the LPSN API but registration is free and easy to accomplish.
